# Quantitative description of upper extremity function and activity of people with spinal muscular atrophy

**DOI:** 10.1186/s12984-020-00757-4

**Published:** 2020-09-11

**Authors:** Mariska M. H. P. Janssen, Laura H. C. Peeters, Imelda J. M. de Groot

**Affiliations:** Department of Rehabilitation, Radboud University Medical Center, Donders Centre for Neuroscience, Reinier Postlaan 4, 6525 GC Nijmegen, the Netherlands

**Keywords:** Spinal muscular atrophy, Upper limb, 3D motion analysis, Surface electromyography, Muscle torque, Range of motion

## Abstract

**Background:**

Therapeutic management of the upper extremity (UE) function of people with spinal muscular atrophy (SMA) requires sensitive and objective assessment. Therefore, we aimed to measure physiologic UE function of SMA patients with different functional abilities and evaluate the relation between these physiologic measures and functional UE scales.

**Methods:**

12 male and 5 female SMA patients (mean age 42 years; range 6–62 years) participated in this explorative study. Concerning the physiologic level, the maximal muscle torque, the maximal and normalized surface electromyography (sEMG) amplitudes, and the maximal passive and active joint angles were measured. Concerning the activity level, the Performance of the Upper Limb (PUL) scale was used, and hand function was examined using the Nine-Hole Peg Test and the Timed Test of In-Hand Manipulation (TIHM).

**Results:**

Outcome measures that significantly related to the functional ability were: the PUL score (all dimensions); the finger to palm task of the Timed TIHM; biceps, triceps, and forearm extensor strength; and the active range of motion of shoulder abduction, shoulder flexion, and wrist extension. In addition, the following physiologic variables were related to the activity level (PUL score): hand function (the Nine-Hole Peg Test; R_s_ = − 0.61), the Timed TIHM (R_s_ = − 0.53), the maximal muscle torque (R_s_ = 0.74), the maximal sEMG amplitude (R_s_ = 0.79), and the maximal active joint angle (R_s_ = 0.88).

**Conclusions:**

Muscle functions in SMA patients are already affected before activity limitations are noticeable. Consequently, monitoring the maximal muscle strength and the normalized muscle activity during task performance could play a role in the early detection of UE limitations. The mechanism behind the loss of arm activities due to SMA is primarily caused by decreasing muscle capacity, which influences the ability to move an arm actively. In clinical practices, these dimensions should be considered separately when monitoring disease progression in order to better evaluate the need for interventions.

## Background

Spinal muscular atrophy (SMA) is an autosomal recessive neuromuscular disorder that involves loss of anterior horn cells in the spinal cord, resulting in muscle weakness and low muscle tone [1]. SMA has an estimated incidence of 1 in 6000 to 11,000 live births [2]. Five types of SMA can be distinguished: type 0, I, II, III and IV [1]. Type 0, or prenatal SMA, is the most severe form of SMA, and children with this type usually die before the age of 6 months. SMA type I is the most common type, and symptoms are present from birth. Children with SMA type I experience severe muscle weakness and are unable to sit without assistance. Without treatment, the life expectancy is about two years. SMA type II is an intermediate form of SMA, where the first symptoms become visible between 6 and 12 months. People with SMA type II initially experience weakness in the proximal muscles, which results in the lifelong inability to ambulate and limited upper extremity (UE) function. SMA type III is a milder form of SMA, in which the first symptoms are present after 2 years of age. People with SMA type III learn to ambulate but often become wheelchair users during their lives. SMA type IV is a rare type of SMA and has an adult onset. SMA type IV patients reach all motor milestones and are affected by the disease relatively mildly [1]. For this study, we will focus on people with SMA type II and SMA type III, as this population can sit, experiences UE involvement and may benefit from interventions that aim to improve UE function. SMA type I patients may also benefit from UE interventions; however, for this population due to severe trunk instability, it is not feasible for them to participate in this study.

In SMA type II and III UE muscle strength is lower in comparison to healthy controls and progressively weakens over time [3–5]. The reduction of UE muscle strength results in difficulties during the performance of UE tasks [6–8], which results in reduced independence, social participation, and quality of life [9, 10]. Hence, the preservation of UE function in persons with SMA is very important.

In order to preserve UE function in people with SMA new interventions that compensate for the loss of UE function are needed. Sensitive, objective assessments are necessary for the development and assessment of these interventions on both a physiological and functional level. By understanding the relation between different aspects of UE function, the underlying mechanisms of declined UE task performance can be unraveled. This mechanism may hold important clues for its treatment [11]. In people with Duchenne muscular dystrophy (DMD), we already started to unravel this mechanism involving declined UE task performance [11, 12]. We found that the decline in muscle function preceded the decline in activity level and that DMD patients use more of their muscle capacity to perform tasks compared with healthy controls. In addition, the active range of motion was strongly related to the ability to perform daily activities. As well, the passive range of motion showed a moderate correlation with both the disease stage and the ability to perform daily activities. Thus, these results give clues for possible interventions like splints, dynamic arm supports, or stretching to prevent specific contractures.

Among the many neuromuscular disorders (NMDs), the underlying cause for the occurrence of muscle weakness that results in functional limitations differs. This variation results in specific UE profiles that apply to different types of NMDs [13]. Consequently, results from one NMD cannot directly be translated to other NMDs; therefore additional research specific to SMA is needed.

The aim of this study is to give a quantitative description of UE functioning during a variety of meaningful UE tasks in people with SMA with different functional abilities. In addition, we aim to evaluate the relation between physiologic UE functions and UE function scales.

## Methods

### Population

17 ambulatory and non-ambulatory people with SMA type II and SMA type III participated in this study. Participants were included if they had a DNA established diagnosis of SMA, were above 6 years of age, and had no comorbidities affecting their arm function. Patients were recruited through the Radboud University Medical Center (Radboudumc) outpatient clinic and by advertisement through the patient organizations “Spierziekten Nederland” and “Prinses Beatrix Spierfonds.” This study was approved by the medical ethical committee of Arnhem–Nijmegen in the Netherlands (Registration number 2016–2824, NL nr.: NL58988.091.16). Informed consent was obtained from all participants and, in the cases where the participants were under 16 years of age, their parents.

### Procedures

#### Participant characteristics

In order to gain more information on factors that might influence the results of this study, we collected the following participant characteristics: SMA type, age at diagnosis, occurrence of scoliosis, UE pain, use of medication, receipt of physical therapy, fatigue (the Visual Analog Scale (VAS) score), hand preference, participation in hobbies, participation in sports, and employment/school information.

#### Setting

All measurements were performed in the movement laboratory of the Radboudumc. Maximal muscle torque and surface electromyography measurements, the Nine-Hole Peg Test, and the Timed Test of In-Hand Manipulation (TIHM) were performed while participants were sitting on a firm chair or, in case participants were non-ambulant, while sitting in their own wheelchair. The performance of upper limb (PUL) scale as well as the active and passive range of motion tasks were performed while sitting on a height adjustable bench without a back support and arm rests in order to examine trunk involvement when performing UE movements [14]. The height of the bench was adjusted so that their feet were flat on the floor and their knees were flexed about 90 degrees. Two of the participants were not able to sit on the bench without any arm and back support and so performed these tasks while sitting in their wheelchair.

#### Functional scales

Following the International Classification for Functioning, Disability, and Health (ICF), functional scales were used to gain more insight into the participant’s activity level to by asking them to perform tasks closely related to daily activities. The scales that we used were: the Nine-Hole Peg Test [15], the Timed TIHM [16], and the PUL scale [17]. All tests were conducted by a trained examiner in a standardized environment. The Nine-Hole Peg Test was performed once with each hand in accordance with the procedures of the National Multiple Sclerosis Society [18]. The total time for each attempt was recorded, and the examiner kept track of the number of pegs that were dropped during the measurement. A low number dropped pegs and a low execution time indicate better hand function. The Timed TIHM was performed twice with the preferred hand in accordance with the procedures described by de Vries et al. [16]. For each of the three tasks (translation from finger to palm, translation from palm to finger, and complex rotation of 360°), we scored the time needed to complete the task and made notes about the number of pegs that were dropped, the participant’s use of the other hand and their sliding of pegs across the surface. Here also, a low execution time and a low number of errors represented better hand function. The PUL items were performed in accordance with the PUL 2.0 user manual [19]. All PUL items were performed once. Based on the score on the entry items, some participants only performed a specific subset of the PUL items. Sum scores of the 3 PUL dimensions (high-level shoulder, mid-level elbow, and distal wrist and hand) and the total score were calculated. A higher score indicated better UE function.

#### Muscle torque

Muscle torque, muscle activity, and 3D motion analysis were used to gain more insight into the ICF level of body functions and structures. Muscle torques of 5 different UE muscles/muscle groups, specifically the trapezius (descending part), the biceps brachii (long head), the triceps brachii (long head), the deltoid (lateral part), and the wrist extensors, were measured in both arms. Muscle torques were measured using a static frame myometer, which consisted of a KAP-E Force Transducer, had a measurement range of 0.2–2000 N (Angewandte System Technik, Dresden, Germany), and had a height-adjustable and position-adjustable frame (custom made at the VU medical center, Amsterdam, the Netherlands). Muscle torques were calculated by multiplying the measured forces of the sensor by the moment arm. Testing positions were based on literature [20, 21],but some muscle testing positions were slightly changed to make measurements feasible for SMA patients who were wheelchair users or had joint contractures. Muscle torque data were filtered using a 3 Hz low-pass Butterworth filter of the 4th order. All participants performed two maximal voluntary isometric contractions (MVICs) to determine the maximal muscle torque. If the maximal values between these two measurements differed by more than 10%, the measurement was repeated. The average maximal value based on the two successful attempts was used for further data analysis.

#### Muscle activity

Wireless sEMG signals of the left and right arm were recorded from the same muscles as were used for the muscle torque measurements with a sample frequency of 1000 Hz (Zerowire EMG, Aurion, Italy). Disk-shaped Ag–AgCL ARBO ECG electrodes (Tyco Healthcare, Neustadt, Germany) were placed at an inter-electrode distance of 24 mm. Electrode positions were based on literature [20, 21]. sEMG data were filtered using a 4th order band-pass filter between 20 and 450 Hz after the signal was rectified and low-pass filtered (3 Hz) to obtain the linear envelope [22, 23]. The maximal muscle activity of each muscle was determined during MVICs; procedures for collecting the MVICs are described above. In addition, normalized sEMG amplitudes were determined during the performance of three daily tasks from the PUL scale, i.e., drinking from a glass that weighs 200 g, moving a 100 g weight across the tabletop, and tracing a path with a pencil. The normalized sEMG amplitude was defined as the maximum sEMG amplitude that was reached during the task as a percentage of the maximal amplitude of the same muscle during MVIC.

#### 3D motion analysis

Three-dimensional motion data were recorded with a sample frequency of 100 Hz using a 10 camera VICON motion analysis system (Oxford Metrics, Oxford, UK). 24 external reflecting markers (ø 14 mm) were placed according to the Upper Limb Model product guide (Revision 1.0 July 2007). 3D kinematics of the wrist, elbow, and shoulder joints were determined using the Upper Limb Model Version 1.0 (© 2007 Vicon Motion Systems Limited). This model was based on the findings of Cutti et al. [24] and Murray et al. [25].

**Fig. 1:**
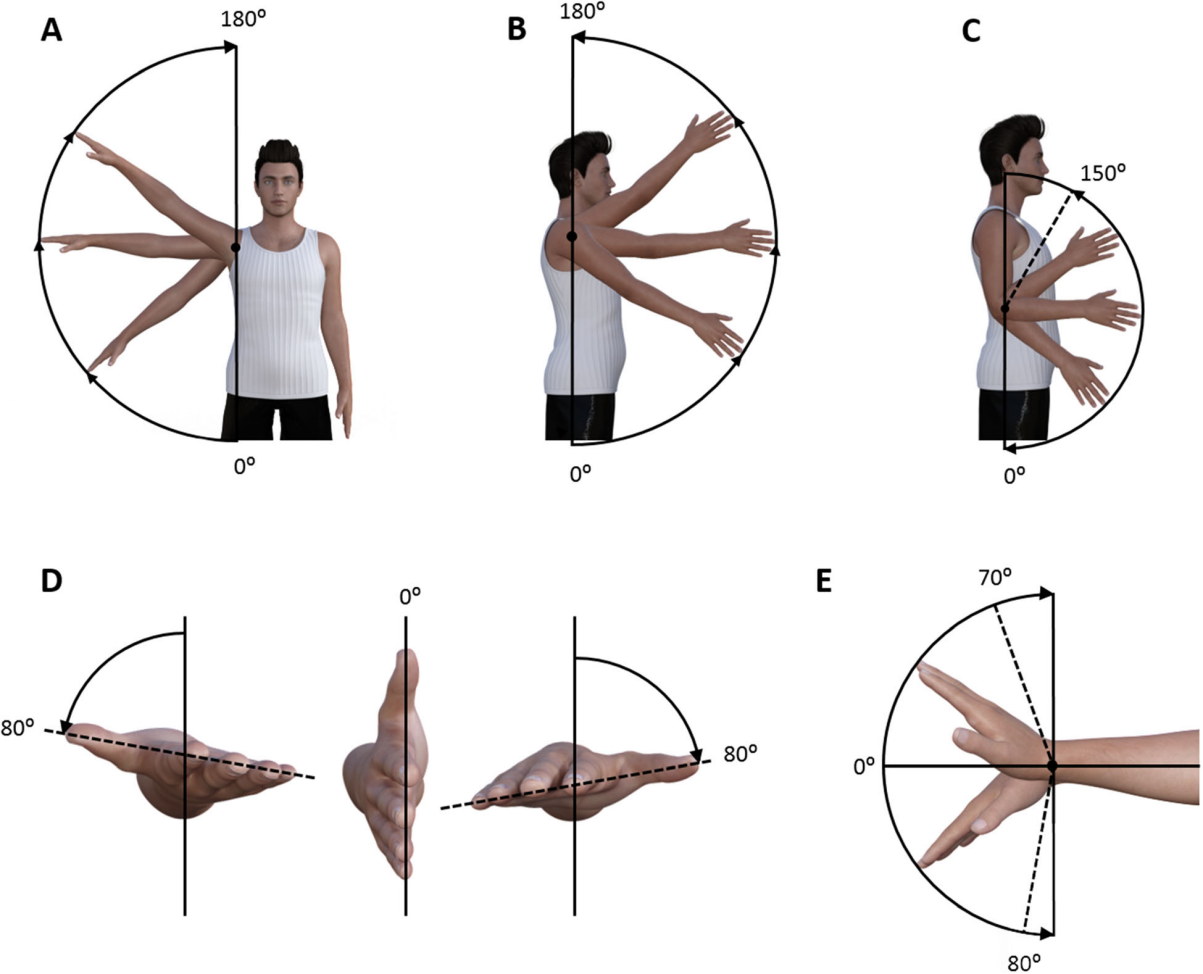
Single joint movements. **a**: shoulder abduction; **b**: shoulder flexion; **c**: elbow flexion and extension; **d**: pronation (right side) and supination (left side); **e**: wrist flexion and extension

3D motion analysis was used to determine the passive (arm moved by the examiner) and active (arm moved by the participant) range of motion for shoulder abduction, shoulder flexion, elbow flexion and extension, pronation and supination of the forearm, and wrist flexion and extension (Fig. 1). All passive and active movements were performed 3 times at a controlled movement velocity (3 s per movement). Kinematic data were filtered using a 4th order low-pass filter of 20 Hz. For each movement, the maximal joint angles were determined. The average maximal joint angle over three measurements was used for further data analysis.

### Data analysis and statistics

Continuous patient characteristics were described using median values and ranges, while categorical data were described using percentages. Mann-Whitney tests were used to test for differences between SMA patients in different categories related to their functional abilities. Categories were based on the Brooke Upper Extremity Rating Scale [26]. Participants in Category A had a Brooke score of 1 or 2 and were able to lift their arms/hands above their head. Participants in Category B had a Brooke score 3 or higher, meaning they were not able to lift their arms/hands above their heads. Classification of UE function based on the Brooke scale has been used before in relation to SMA patients [5, 27]. We chose to combine the Brooke scores of 1 and 2 into one category and the Brooke scores 3–6 into another category because the ability to lift the arm overhead (thus shoulder level activities are possible) is of clinical importance and is often used as a milestone for considering interventions.

To gain insight into the relation between functional UE scales (the PUL scale, the Nine-Hole Peg Test, and the Timed TIHM) and physiologic UE function (muscle torque, muscle activity, and passive and active joint angles), we calculated Spearman correlation coefficients between the coupled total scores of these outcome measures. Correlations between 0.3 and 0.5, between 0.5 and 0.7, between 0.7 and 0.9, and over 0.9 were interpreted as low, moderate, high, and very high, respectively [28]. The total scores were calculated by adding the results of all values within one outcome measure, i.e., the sum of the individual items. If one or more values were missing, the total score was also reported as missing. If values were missing because patients were physically unable to perform the activity, a score of 0 was used for the calculation of the total scores. Data were processed with custom-written Matlab (Matlab® version R2014b, Mathworks, Natick, USA) routines. SPSS Statistics Version 20 (IBM, Somers, USA) was used for statistical analysis.

## Results

Of the total 17 SMA patients who participated in this study, 12 were male, and 5 were female. The median age was 42 years. The age range was 6–62 years. 6 of the participants were diagnosed with SMA type II (35%), while 11 participants were diagnosed with SMA type III (65%). 15 patients (88%) were non-ambulant, and the median Brooke score was 3 (range of 1–6). 9 of the participants had scoliosis (53%), which was surgically corrected in 6 participants (67%). 1 of the participants used Pyridostigminebromide (Mestinon®). This patient, however, did not present itself as an outlier. Table 1 provides an overview of individual participant characteristics.

**Table 1:** Participant characteristics

Subject	Age (years)	Gender	SMA type	Age at diagnosis (years)	Scoliosis	Scoliosis surgery	Pain^a^	Vignos	Brooke
001	48	Male	3	28	No	n.a.	No	2	1
002	61	Male	3	34	No	n.a.	Yes	1	3
003	32	Male	3	15	No	n.a.	Yes	9	2
004	61	Male	3	20	Yes	No	No	9	3
005	58	Male	3	17	No	n.a.	No	9	3
007	62	Male	2	1	Yes	Yes	No	9	6
008	19	Male	3	2	Yes	No	No	9	3
009	15	Male	2	2	Yes	Yes	No	9	3
010	44	Male	3	11	No	n.a.	No	9	2
011	6	Male	2	2	No	n.a.	No	9	1
012	16	Female	2	1	Yes	Yes	No	9	3
013	35	Female	3	2	Yes	No	Yes	9	1
014	43	Female	2	2	Yes	Yes	No	9	5
017	57	Female	3	4	No	n.a.	Yes	9	1
018	32	Female	3	3	Yes	Yes	No	9	2
019	51	Male	3	10	No	n.a.	Yes	9	3
020	20	Male	2	2	Yes	Yes	No	9	4

^a^ Experiences pain at back, shoulder(s) or arm(s) at time of measurement. SMA = spinal muscular atrophy, n.a. = not applicable

**Fig. 2:**
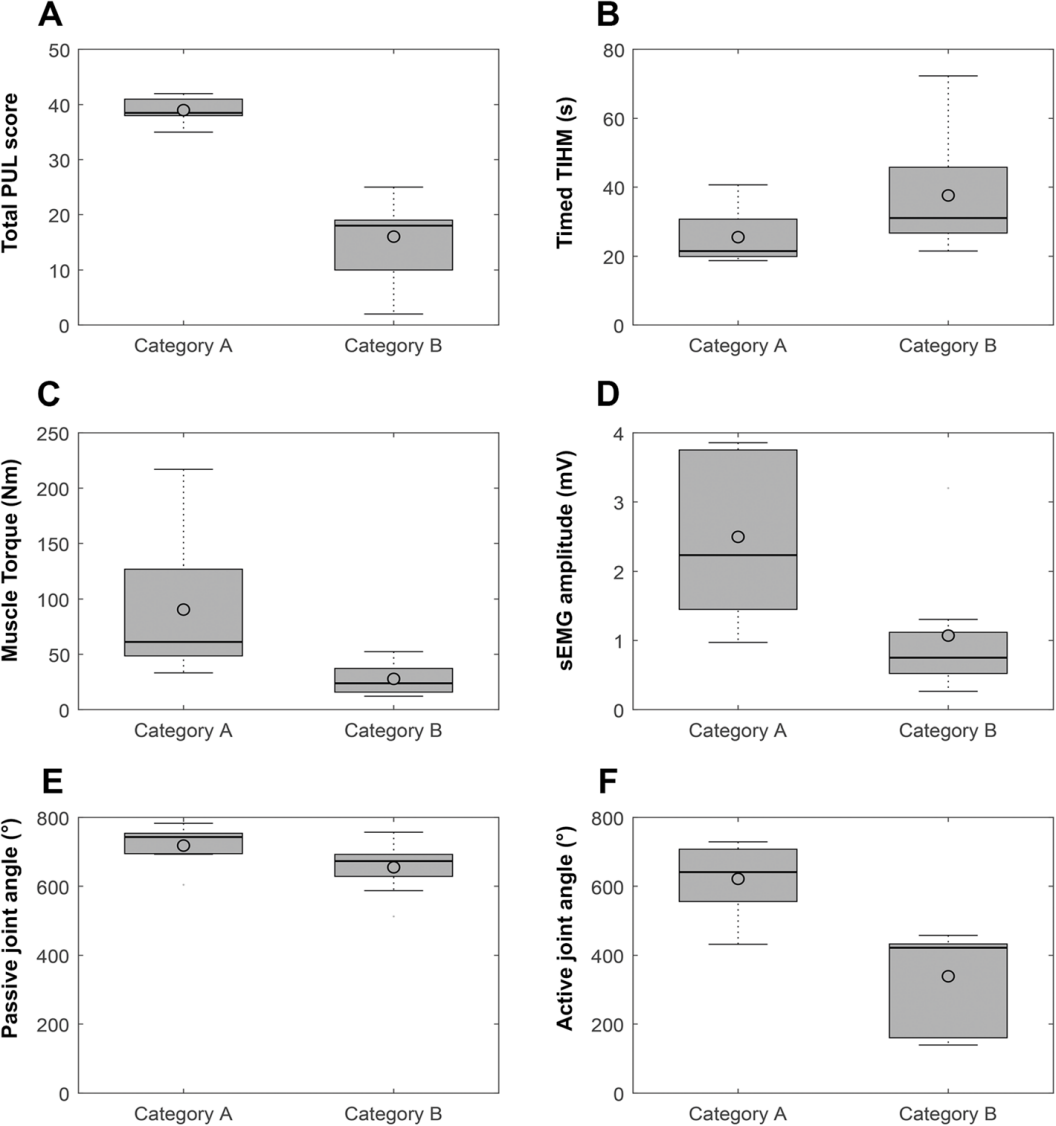
Change over disease categories. **a**: total PUL score; **b**: Timed TIHM; **c**: maximal muscle torque; **d**: maximal sEMG amplitude; **e**: passive maximal joint angle; **f**: active maximal joint angle

Table 2 shows an overview of the UE function of SMA patients for all outcome variables. Next to the median scores of the entire population (the total group), the median scores per functional category are displayed. A higher category indicates a worse functional ability. Significant differences between categories were seen for the PUL total score (*p* < 0.001), the maximal muscle torque total score (*p* = 0.015), the maximal sEMG amplitude total score (*p* = 0.032), the maximal active range of motion total score (*p* = 0.014), and the Nine-Hole Peg Test total score (*p* = 0.012). When performing the Nine-Hole Peg Test, 59% of the participants made an error, i.e., dropped or slid one or more of the pegs. 88% of the participants made at least 1 error during the performance of the Timed TIHM. Figure 2 shows boxplots for the total scores of each outcome measure per functional category.

**Table 2:** Upper extremity function of SMA patients per category

		Total group			Category A^b^			Category B			
	Side	N	Mean	95% CI	N	Mean	95% CI	N	Mean	95% CI	*p*-value Category
**Performance of upper limb scale**^a^											
High level shoulder dimension		17	8	(0;12)	8	8,5	(5;12)	9	0	(0;1)	**< 0,001**
Mid level elbow dimension		17	10	(1;20)	8	19	(19;20)	9	8	(1;15)	**< 0,001**
Distal wrist and hand dimension		17	11	(1;11)	8	11	(10;11)	9	9	(1;11)	**0,005**
Total score		17	27	(2;42)	8	38,5	(35;42)	9	18	(2;25)	**0,001**
**Nine-hole peg test (s)**											
Time to complete	Dominant	16	25.4	(17.2;72.3)	8	23,0	(17,2;27,8)	8	30,6	(21,6;72,3)	0,156
Time to complete	Non-dominant	16	24.9	(21.0;76.0)	8	22,5	(21,0;26,4)	8	33,9	(23,3;76,0)	**0,003**
Total score		16	49.9	(39.7;138.0)	8	46,5	(39,7;52,9)	8	61,8	(48,1;138,0)	**0,012**
**Timed TIHM (s)**											
Finger to palm task		16	3.8	(2.7;18.0)	8	3,2	(2,7;3,8)	8	4,7	(3,1;18,0)	**0,003**
Palm to finger task		16	9.8	(5.7;27.9)	8	8,8	(5,7;19,3)	8	12,1	(6,2;27,9)	0,208
Complex rotation task		16	13.0	(7.3;33.0)	8	10,1	(7,3;21,4)	8	14,2	(11,0;33,0)	0,093
Total score		16	28.3	(18.7;72.3)	8	21,5	(18,7;40,7)	8	31,0	(21,5;72,3)	0,074
**Maximal muscle torque (Nm)**											
Trapezius	Dominant	17	20	(3;90)	8	25	(8;90)	9	12	(3;36)	0,083
Trapezius	Non-dominant	17	20	(1;88)	8	24	(10;88)	9	13	(1;36)	0,083
Biceps	Dominant	15	6	(0;65)	8	10	(5;65)	7	3	(0;6)	**0,002**
Biceps	Non-dominant	15	5	(1;58)	6	13	(3;58)	9	3	(1;9)	**0,013**
Triceps	Dominant	17	3	(1;35)	8	9	(4;35)	9	2	(1;3)	**0,001**
Triceps	Non-dominant	15	2	(1;54)	6	8	(2;55)	9	1	(1;3)	**0,003**
Deltoid	Dominant	17	9	(1;38)	8	13	(7;38)	9	4	(1;17)	**0,012**
Deltoid	Non-dominant	15	9	(1;65)	6	10	(7;65)	9	5	(1;21)	0,099
Forearm extensors	Dominant	16	3	(0;12)	8	5	(2;12)	8	1	(0;5)	**0,009**
Forearm extensors	Non-dominant	14	3	(0;12)	6	4	(2;12)	8	2	(0;5)	**0,028**
Total score	Dominant	15	45	(12;217)	8	61	(33;217)	7	24	(12;52)	**0,005**
Total score	Non-dominant	14	39	(5;277)	6	56	(35;277)	8	29	(5;55)	**0,028**
Total score		13	86	(35;433)	6	104	(68;433)	7	56	(35;108)	**0,015**
**Maximal sEMG amplitude (mV)**											
Trapezius	Dominant	17	0.15	(0.02;0.72)	8	0,23	(0,07;0,51)	9	0,11	(0,02;0,72)	0,124
Trapezius	Non-dominant	17	0.23	(0.05;1.36)	8	0,25	(0,05;1,36)	9	0,20	(0,05;1,04)	0,630
Biceps	Dominant	15	0.16	(0.04;0.90)	7	0,27	(0,07;0,90)	8	0,13	(0,04;0,58)	**0,049**
Biceps	Non-dominant	15	0.13	(0.01;1.01)	6	0,46	(0,03;1,01)	9	0,10	(0,01;0,28)	0,099
Triceps	Dominant	15	0.10	(0.02;1.65)	8	0,20	(0,04;1,65)	7	0,08	(0,02;0,38)	0,118
Triceps	Non-dominant	14	0.09	(0.02;0.71)	6	0,32	(0,03;0,71)	8	0,06	(0,02;0,22)	0,071
Deltoid	Dominant	17	0.18	(0.03;1.11)	8	0,47	(0,03;1,11)	9	0,11	(0,03;0,86)	0,083
Deltoid	Non-dominant	15	0.20	(0.03;1.49)	6	0,73	(0,08;1,49)	9	0,11	(0,04;0,70)	**0,021**
Forearm extensors	Dominant	16	0.48	(0.08;1.58)	8	0,70	(0,36;1,58)	8	0,24	(0,09;0,81)	**0,012**
Forearm extensors	Non-dominant	14	0.26	(0.11;0.58)	6	0,37	(0,21;0,58)	8	0,21	(0,11;0,31)	**0,028**
Total score	Dominant	14	1.37	(0.26;3.86)	7	2,23	(0,97;3,86)	7	0,75	(0,27;3,20)	**0,013**
Total score	Non-dominant	14	1.45	(0.36;3.76)	6	2,32	(0,60;3,76)	8	0,78	(0,36;2,19)	**0,020**
Total score		13	3.50	(0.98;7.62)	6	5,16	(1,58;7,62)	7	1,61	(0,98;5,25)	**0,032**
**Passive maximal joint angle (°)**											
Shoulder abduction	Dominant	14	138	(76;157)	7	149	(132;157)	7	130	(76;146)	**0,018**
Shoulder abduction	Non-dominant	14	137	(109;161)	7	146	(123;150)	7	118	(109;161)	0,064
Shoulder flexion	Dominant	15	149	(78;168)	8	162	(136;168)	7	139	(78;158)	**0,011**
Shoulder flexion	Non-dominant	16	153	(103;173)	8	156	(126;173)	8	125	(103;163)	0,059
Elbow flexion	Dominant	17	151	(142;168)	8	148	(142;161)	9	153	(144;162)	0,193
Elbow flexion	Non-dominant	17	150	(95;167)	8	149	(145;163)	9	152	(95;168)	0,246
Elbow extension	Dominant	17	23	(4;98)	8	19	(4;32)	9	28	(7;90)	0,178
Elbow extension	Non-dominant	17	24	(− 84;90)	8	23	(5;42)	9	24	(−84;98)	0,962
Pronation	Dominant	17	97	(72;134)	8	97	(72;106)	9	97	(26;120)	0,594
Pronation	Non-dominant	17	74	(26;106)	8	72	(50;106)	9	79	(48;134)	0,470
Supination	Dominant	17	−72	(−99;22)	8	−66	(−99;-44)	9	−85	(−109;-65)	**0,034**
Supination	Non-dominant	17	− 101	(− 131;-64)	8	−96	(−103;-64)	9	−109	(−131;22)	0,149
Wrist flexion	Dominant	17	−80	(−89;-25)	8	−78	(−85;-70)	9	−80	(−89;-25)	0,664
Wrist flexion	Non-dominant	16	−83	(−97;-47)	8	−83	(−88;-58)	8	−82	(−97;-28)	0,599
Wrist extension	Dominant	17	56	(33;78)	8	51	(33;56)	9	62	(49;78)	**0,018**
Wrist extension	Non-dominant	16	52	(30;77)	8	47	(30;57)	8	60	(42;77)	**0,012**
Total score	Dominant	14	694	(513;783)	7	743	(604;783)	7	673	(534;757)	0,085
Total score	Non-dominant	14	721	(534;796)	7	722	(585;796)	7	707	(513;792)	0,848
Total score		13	1425	(1046;1579)	7	1469	(1189;1579)	6	1348	(1046;1549)	0,253
**Active maximal joint angle (°)**											
Shoulder abduction	Dominant	17	32	(0;164)	8	144	(32;164)	9	24	(0;32)	**0,001**
Shoulder abduction	Non-dominant	15	29	(0;156)	6	133	(50;156)	9	18	(0;30)	**0,001**
Shoulder flexion	Dominant	16	52	(0;180)	8	144	(53;180)	8	8	(0;52)	**0,001**
Shoulder flexion	Non-dominant	16	34	(0;167)	7	113	(31;167)	9	14	(0;45)	**0,002**
Elbow flexion	Dominant	17	144	(0;166)	8	143	(135;159)	7	144	(50;159)	0,907
Elbow flexion	Non-dominant	16	146	(48;162)	7	146	(133;162)	9	149	(48;166)	0,916
Elbow extension	Dominant	15	25	(10;98)	8	22	(10;33)	7	30	(21;92)	0,081
Elbow extension	Non-dominant	16	27	(9;114)	7	21	(9;51)	9	29	(12;114)	0,204
Pronation	Dominant	17	72	(33;110)	8	73	(70;110)	9	70	(−5;90)	0,111
Pronation	Non-dominant	16	58	(−45;89)	7	54	(16;89)	9	67	(−45;89)	0,525
Supination	Dominant	17	−52	(−92;68)	8	−50	(−86;-20)	9	−63	(−93;16)	0,312
Supination	Non-dominant	16	−78	(−104;-48)	7	−79	(−104;-52)	9	−67	(−92;68)	0,560
Wrist flexion	Dominant	16	−68	(−77;39)	8	−70	(−77;-46)	8	−64	(−72;39)	0,115
Wrist flexion	Non-dominant	15	−70	(−84;7)	7	− 78	(−84;-52)	8	−66	(−83;20)	0,385
Wrist extension	Dominant	16	48	(28;67)	8	44	(28;52)	8	55	(44;67)	**0,011**
Wrist extension	Non-dominant	15	52	(8;65)	7	40	(8;52)	8	55	(54;65)	**0,001**
Total score	Dominant	14	506	(139;729)	8	641	(432;729)	6	422	(139;458)	**0,005**
Total score	Non-dominant	14	438	(246;726)	6	623	(389;726)	8	393	(160;469)	**0,010**
Total score		12	916	(432;1456)	6	1236	(948;1456)	6	836	(432;884)	**0,004**

^a^Performance of Upper Limb (PUL 2.0) scale (maximal score = 42)

^b^Categories are based on Brooke upper extremity rating scale in combination with the score on the Performance of Upper Limb scale. Category A: Brooke 1 and 2, good midlevel and distal PUL score. Category B: Brooke 3–6, moderate to poor midlevel and moderate to good distal PUL score

*Significant *p*-value is 0.05

**Fig. 3:**
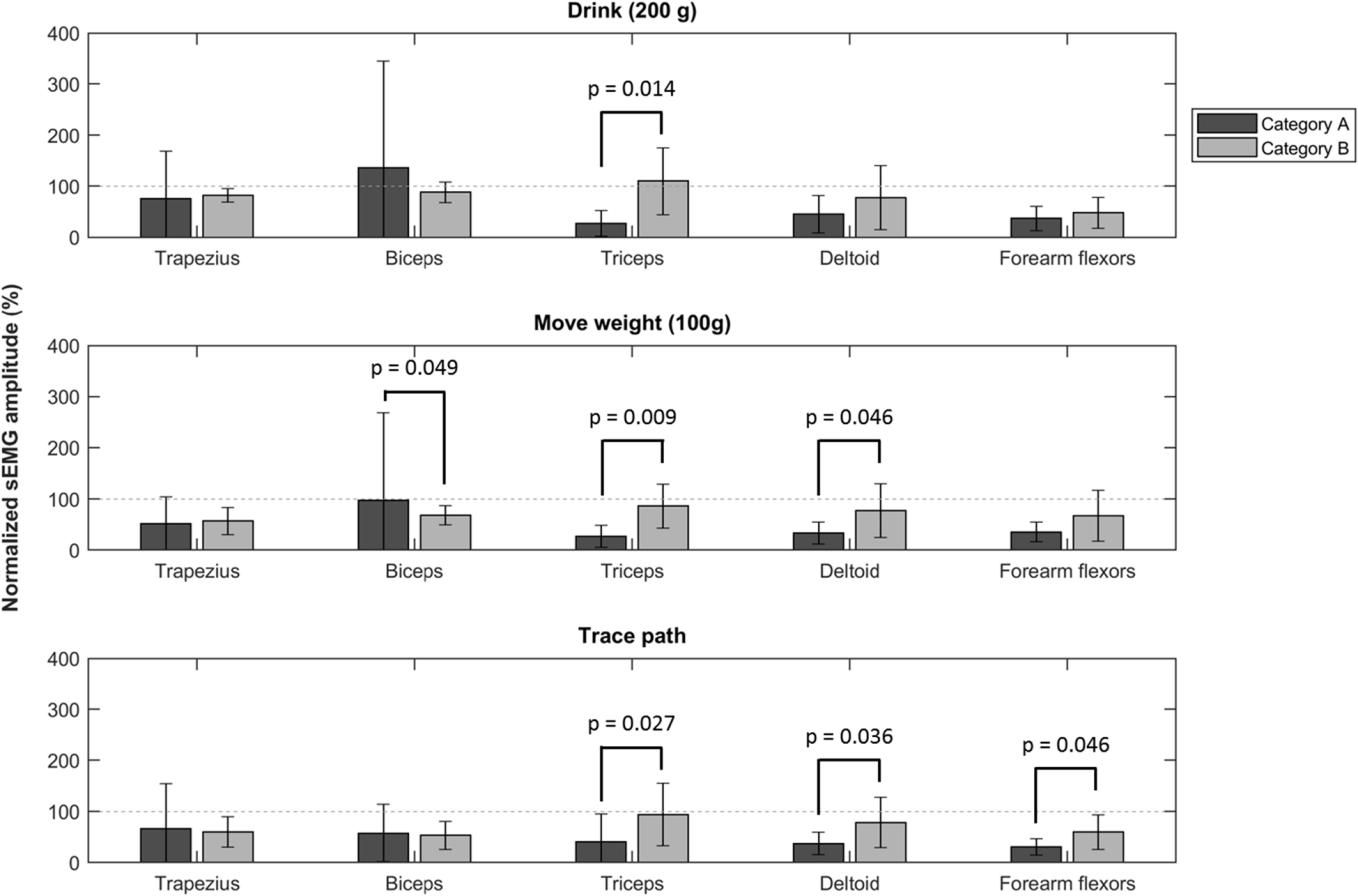
Normalized sEMG amplitudes. Normalized sEMG amplitudes of the Trapezius, Biceps Brachii, Triceps Brachii, Deltoid, and forearm extensor muscles for 3 different upper extremity movements shown for different disease categories. A: drinking from a glass filled with 200 g; B: moving a 100 g weight over the tabletop; C: tracing a path with a pencil. Note: Drinking from a glass filled with 200 g could not be performed by the participants in category 3

Figure 3 shows normalized sEMG amplitudes of 5 UE muscles of the dominant arm during drinking, moving a weight, and tracing a path with a pencil. There is a large variability among participants, which is mainly caused by several participants showing extreme sEMG amplitudes (> 100% MVIC). In addition, normalized sEMG values for all muscles reach values close to or over 100% MVIC. Significant differences between functional categories were found for the triceps during drinking (*p* = 0.014), moving a weight (*p* = 0.009) and during tracing a path (*p* = 0.027), the deltoid during moving a weight (*p* = 0.046) and during tracing a path (*p* = 0.036), the biceps during moving a weight (*p* = 0.049), and the forearm extensors during tracing a path (p = 0.046). It should be noted that some participants in Category B were not able to drink from a glass filled with 200 g (PUL 2.0, item 7).

Table 3 shows the Spearman correlations between outcome measures and total scores. The PUL total score shows a high correlation with the maximal muscle torque total score (r_s_ = 0.74), with the maximal sEMG amplitude total score (r_s_ = 0.79), and with the active maximal joint angle total score (r_s_ = 0.88). The PUL total score correlates moderately with the Nine-Hole Peg Test total score (r_s_ = − 0.61), and the Timed TIHM total score (r_s_ = − 0.53). In addition, the Nine-Hole Peg Test total score is moderately correlated with the Timed TIHM total score (r_s_ = 0.66) and with the active maximal joint angle total score (r_s_ = − 0.67). The maximal sEMG amplitude shows a high correlation with the active maximal joint angle total score (r_s_ = 0.79). Lastly, the passive maximal joint angle total score shows a moderate correlation with the active maximal joint angle total score (r_s_ = 0.64).

**Table 3:** Spearman correlation coefficients of total scores

	1	2	3	4	5	6	7
1. PUL score	1,00						
2. Nine-hole peg test	−0,61*	1,00					
3. Timed TIHM	−0,53*	0,66**	1,00				
4. Maximal muscle torque	0,74**	−0,42	− 0,63*	1,00			
5. Maximal sEMG amplitude	0,79**	−0,31	0,06	0,55	1,00		
6. Passive maximal joint angle	0,38	−0,38	−0,29	0,09	0,25	1,00	
7. Active maximal joint angle	0,88**	−0,67*	− 0,38	0,62	0,79**	0,64*	1,00

The total scores were calculated by adding the results of all values within one outcome measure (sum of individual items)

* Statistical significant correlation (*p*-value < 0.05) ** Statistical significant correlation (*p*-value < 0.01)

## Discussion

This study provides new insights into UE function and the activity level of people with SMA type II and type III. It offers a quantitative description of different aspects of UE function based on the ICF body function and structure level, and activity level. Individual results regarding the UE function at different functional stages are comparable to previous reported results. However, this study provides novel insights into the relation between different aspects of UE function. We described UE function using objective outcome measures like 3D motion analysis and (normalized) surface electromyography. These insights can be used for clinical decision-making and for the development and evaluation of new interventions.

### Declined task performance

By displaying the results over 2 functional categories with increasing limitations in arm activity due to increasing disease severity (Table 2 and Fig. 2), we can give a general estimation of the disease progression for individual variables. As expected, we saw that both functional (physiological) ability and the ability to perform activities were lower in Category B (worse functional ability). Nevertheless, there is a large variability among participants in the different functional categories, especially with regard to the ability to generate muscle torque and muscle activity levels.

The muscle strength of all muscles, except the trapezius muscle, declined with the functional category. Participants in Category A showed an almost normal ability to perform activities, while their maximal muscle strength was already much lower compared to reference values of healthy persons [29–32]. This result indicates that reduced muscle torque does not directly lead to the inability to perform daily activities, which may be due to the fact that the participants’ maximum muscle torque is still higher than the required torque to perform a task. Another explanation could be the fact that people with SMA use a lot of compensatory mechanisms for task performance [33]. The compensatory strategies that we saw during the measurements were: reducing the joint torque during shoulder abduction by bending the elbow, using swinging movements to gain enough momentum to move the arm, using trunk movements to accelerate the arm, and activating secondary muscles to increase the power output. In fact, the relatively high levels of normalized sEMG amplitudes in the various muscles that we have measured in this study could be an indicator of the use of secondary muscles during task performance. These findings indicate that the measuring of muscle strength and muscle activity could play a role in the early detection of UE limitations. In particular, high levels of normalized muscle activity could be predictive of the loss of function since a function is lost when the maximal muscle capacity (100% of MVIC) is reached. In addition, the use of high levels of normalized muscle activity could lead to a faster occurrence of fatigue [34], which is a common symptom seen in people with SMA [35, 36].

A decline of muscle torque was present in the proximal (deltoid), mid-level (biceps and triceps), and distal (wrist extensors) muscles. Especially torques produced by the triceps are very low in Category B (range 1–3 Nm for both arms). This result is in line with literature, where it is stated that the triceps is one of the muscles that is affected early on in people with SMA [37, 38]. Our study also shows low torque values for the biceps, deltoid, and forearm extensor muscles, which are reported to be better preserved in people with SMA [37]. This difference might be due to the method of strength measurement. Wademan et al. used the medical research council (MRC) scale to categorize muscle strength, while we measured muscle strength in absolute values (N) using a static frame. It is possible that persons can still obtain an MRC level of 4 or 5 (able to move against resistance to normal force) while the maximal absolute strength level has already reduced significantly. Regarding normalized sEMG levels during task performance, the triceps muscle shows a difference in functional category for all three tasks. In addition, normalized sEMG levels of the triceps muscle are very close to 100% MVIC during task performance. This finding is also in line with the fact that the triceps muscle is relatively weak and affected early on in people with SMA [37, 38]. Participants in Category B show high levels of normalized muscle activity for all muscles (> 50%), meaning that all UE muscles are affected and performing close to their maximal capacity during the performance of daily life tasks in persons with SMA with a relatively low functional ability (>Brooke 3).

Concerning the active range of motion, we see that mainly the range of motion in the shoulder is impaired in SMA patients. The limitations in this range of motion, in contrast to muscle torque, only occur in functional Category B as close to normal values are seen in the patients within Category A. Passive range of motion does not significantly decline with functional ability, although passive range of motion in the shoulder tends to decrease in Category B. In comparison to other neuromuscular disorders such as Duchenne muscular dystrophy (DMD), less contractures seem to be present in the UE of persons with SMA [12, 39]. Despite the difference in the presence of contractures, UE splints are prescribed to both groups. For people with DMD, splints are mainly used for the prevention of contractures that occur as a result of the imbalance between agonist and antagonist muscles [40]. For people with SMA, splints are used to support unstable and hypermobile joints that result from general muscular weakness [35]. Yet, splints for the arms and hand are less commonly prescribed in people with SMA (2%) compared to people with DMD (9.4%) [41, 42].

### Relation UE function and activity

We also examined the relation between activity level and physiologic UE functions to gain insight into the role of different physiologic functions on task performance. The maximal active joint angle, maximal muscle torque, and maximal sEMG amplitude showed high correlations with the activity level, indicating that both the ability to use the full range of motion as well as sufficient muscle strength and activation are needed for adequate task performance. Close monitoring of both muscle function and active range of motion could, therefore, play an important role in starting interventions that minimize functional UE decline in a timely manner. Also, we found a moderate correlation between the PUL score and hand function (i.e., the Nine-Hole Peg Test and Timed TIHM), indicating that both scales measure different aspects of UE function and therefore should be examined separately in clinical practices. This finding is in line with the findings of de Vries et al.,. who found a moderate correlation between the Nine-Hole Peg Test and the Timed TIHM and stated that the tests measure different aspects. They found that the Timed-TIHM evaluates complex patterns, including hand manipulation skills and that the Nine-Hole Peg Test evaluates simple patterns of fine motor coordination, e.g., picking up, placing, and releasing pegs [16]. The occurrence of contractures, i.e., decreased passive range of motion, was relatively uncommon. Hence, it is likely that contractures are not the limiting factor for task performance, which also reflects the fact that we did not find a relation between passive range of motion and UE activity level. In fact, the lack of contractures and occurrence of hypermobility in people with SMA could even have a negative effect on task performance [35]. Although contractures limit the range of motion, they result from tissue changes due to inactivity and muscle weakness, e.g., the infiltration of fatty and connective tissue in the muscle. The stiffening of the tissue, especially at the end range of motion, may allow for better energy storing capacity and the ability to more functionally position the joints [43].

Another aspect that should not be forgotten in this context is the involvement of the trunk in the ability to perform seated daily activities. The ability to perform UE tasks is not only dependent on arm strength and range of motion but also on how well a person is able to use the trunk when performing tasks [14]. Based on the results of this study and additional literature, we can conclude that the ability to perform UE tasks is dependent on many different factors and that the contribution of these factors differ between individuals with SMA. Therefore, we recommend to base treatments/interventions aimed at the improvement of UE limitations on the characteristics and needs of individuals, instead of using a one-size-fits-all approach.

### Study limitations

A limitation of this study is that the study population is relatively small. A very heterogeneous group of 17 participants was included, which allowed us to examine different aspects of UE function in relation to disease severity. However, due to the large variation in both disease severity and age of the participants, the results should be interpreted with care. The cross-sectional nature of this study did not give insight into the longitudinal decline of UE function. For future research, we would recommend a longitudinal analysis of UE function across all dimensions of the ICF (including participation) in persons with SMA. Nevertheless, we believe our population to be a good representation of the general SMA type II and SMA type III population.

Another limitation is the relatively large amount of missing data, especially regarding the calculation of the sum scores. On average, the data of only 13 participants could be used for the calculation of sum scores and thus further analysis. The reason for the missing data was twofold. First, the participants with more severe limitations were not able to perform all the measurements correctly, for example, since they were not able to maintain the starting position during strength measurements. Second, we adapted the measurement protocol to include both arms instead of one arm after measuring two participants because we saw large variability between the left and right hand for those participants.

Regarding the measurement of muscle torques and normalized sEMG amplitudes, there are also some limitations. Muscle torques of individual muscles were reported; however, the co-contraction of other muscles might have influenced these torque measurements. The effect of co-contraction was minimized by using measurement positions that mainly required activation of the prime mover. Maximal sEMG amplitudes (in MVIC) can be influenced by pain, the fear of pain, restrictions in the range of motion, and/or motivation, and so the maximal sEMG amplitude and force can potentially be underestimations [21]. In addition, the dynamics of the movements (velocity and acceleration) performed could have altered the sEMG amplitude during dynamic tasks [44]. When compensatory, swinging movements are used during task performance, this dynamic may have resulted in higher sEMG amplitudes. As a result, normalized sEMG amplitudes over 100% MVIC were sometimes seen. For future studies, we recommend to standardize the movement velocity of dynamic tasks and to record pain intensity and fatigue scores when performing MVIC measurements for better interpretation of the results.

Despite these limitations, we think this study gives valuable and objective insights into UE function and activity level of people with SMA that are of great clinical importance for the selection and evaluation of suitable interventions.

### Clinical implications

We showed that muscle torque is already affected when function ability is still relatively well preserved. Therefore, muscle torque measurements could be used to detect UE decline early. In addition, we saw that contractures are not very common in people with SMA; therefore, measuring passive range of motion (especially at an early stage of the disease) seems redundant. We also saw that persons with SMA use relatively high levels of muscle activity during task performance which can be related to fatiguability. Hence, the assessment of (muscle) fatigue in clinical practice already at an early stage of the disease may be beneficial and allow for early clinical intervention. Interventions that may reduce fatigue and improve UE function are dynamic arm supports. We recommend discussing the use of an arm support in the early phase of the disease because we saw much functional decline in participants with Brooke 3 or higher. Starting interventions early on may lead to the reduction of disuse. Finally, the results of this study can be useful for the development of arm exoskeletons, specifically for setting the torque requirements, determining the necessary range of motion, and determining in which phase passive or actuated support is needed.

## Conclusions

Muscle function in SMA patients are already affected before activity limitations are noticed. As a result, measurements of muscle function could play a role in the early detection of UE limitations. Measurements of maximal muscle strength and normalized muscle activity during task performance are important indicators for the ability to perform daily activities and the effort that is required to perform these activities, which is related to fatiguability. Activity level shows the strongest correlation with active joint angle and muscle strength, while passive joint angle, i.e. contractures, shows the lowest correlation. The mechanism behind the gradual loss of arm activities in SMA patients is thus mostly likely determined by a decreasing of muscle capacity, which influences the possibility to move the arm actively. In clinical practices, these dimensions should be considered separately when monitoring disease progression in order to evaluate the need for interventions, such as splints and arm supports.

## Data Availability

The datasets used and/or analyzed during the current study are available from the corresponding author upon reasonable request.

## Abbreviations

DMD: Duchenne Muscular Dystrophy
ICF: International Classification for Functioning, Disability and Health
MRC: Medical Research Counsel
MVIC: Maximal voluntary isometric contraction
NMD: Neuromuscular disorders
PUL: Performance of Upper Limb
sEMG: surface electromyography
SMA: Spinal Muscular Atrophy
UE: Upper Extremity
TIHM: Test of In-Hand Manipulation
VAS: Visual Analog Scale
